# Quantitative Models of *In Vitro* Bacteriophage–Host Dynamics and Their Application to Phage Therapy

**DOI:** 10.1371/journal.ppat.1000253

**Published:** 2009-01-02

**Authors:** Benjamin J. Cairns, Andrew R. Timms, Vincent A. A. Jansen, Ian F. Connerton, Robert J. H. Payne

**Affiliations:** 1 School of Biological Sciences, University of Bristol, Bristol, United Kingdom; 2 Division of Food Sciences, University of Nottingham, Sutton Bonington Campus, Loughborough, United Kingdom; 3 School of Biological Sciences, Royal Holloway University of London, Egham, Surrey, United Kingdom; Massachusetts General Hospital, United States of America

## Abstract

Phage therapy is the use of bacteriophages as antimicrobial agents for the control of pathogenic and other problem bacteria. It has previously been argued that successful application of phage therapy requires a good understanding of the non-linear kinetics of phage–bacteria interactions. Here we combine experimental and modelling approaches to make a detailed examination of such kinetics for the important food-borne pathogen *Campylobacter jejuni* and a suitable virulent phage in an *in vitro* system. Phage-insensitive populations of *C. jejuni* arise readily, and as far as we are aware this is the first phage therapy study to test, against *in vitro* data, models for phage–bacteria interactions incorporating phage-insensitive or resistant bacteria. We find that even an apparently simplistic model fits the data surprisingly well, and we confirm that the so-called inundation and proliferation thresholds are likely to be of considerable practical importance to phage therapy. We fit the model to time series data in order to estimate thresholds and rate constants directly. A comparison of the fit for each culture reveals density-dependent features of phage infectivity that are worthy of further investigation. Our results illustrate how insight from empirical studies can be greatly enhanced by the use of kinetic models: such combined studies of *in vitro* systems are likely to be an essential precursor to building a meaningful picture of the kinetic properties of *in vivo* phage therapy.

## Author Summary

Phage therapy is an antimicrobial treatment based on specific viruses which are natural predators of bacteria. This approach is being promoted as a possible alternative treatment for use against antibiotic-resistant strains of bacteria. Despite its long history and many potential benefits, adoption of phage therapy has been retarded by a variety of factors, including a poor understanding of the therapeutic consequences of the phage–bacteria relationship. In our work we bring together theory and data by testing kinetic models of phage–bacteria interactions against data for an important agent of human food poisoning, *Campylobacter jejuni*. Our model explicitly allows for resistant bacteria because these have not been properly accounted for in previous phage therapy theory but will be relevant to practical applications. The excellent fit of our model to the data confirms the value of such combined approaches and supports an interpretative viewpoint based on critical density-dependent thresholds that are not part of standard pharmacology. We also find that phage activity appears to be dose-dependent, and we speculate on possible causes for this. Our work illustrates how mathematical models can considerably enhance insights from empirical studies, as an important step in advancing the understanding of phage therapy.

## Introduction

The problem of antibiotic resistance has rapidly increased in recent years. Diseases that had previously been well-controlled are again becoming serious threats to animal and public health in a variety of contexts [1]–[3]. The recognition of antibiotic resistance as a major health problem has led to renewed interest in alternative antimicrobial therapies, including bacteriophage therapy [4]–[6]. Phage therapy has many potential advantages over traditional antibiotics, including specificity for the target organism, an apparent lack of toxicity or immunogenicity associated with lytic phage [7], and the relative ease with which naturally occurring lytic phages can be isolated against particular organisms and produced in quantity. (Phage therapy has steered clear of using temperate phages due to their potential for carrying toxin or antibiotic-resistance genes.) Despite these advantages, development and adoption of phage therapy has been slow. Outside of some former Eastern Bloc countries, few phages have been developed as practical antimicrobials since the widespread adoption of antibiotics [4], although there are some recent exceptions, such as the Food and Drug Administration (FDA) approval in 2006 of a cocktail of six phages for the control of *Listeria monocytogenes* on ready-to-eat meats [8].

One reason for the slow implementation of phages as antimicrobial agents might be that the paradigms associated with antibiotic therapies cannot easily be transferred to phage therapies. For instance, it has been predicted that certain threshold phenomena, not normally encountered in the pharmacokinetics and pharmacodynamics (PK/PD) of antibiotic therapies, are of central importance in practical phage therapy [9]–[11]. It is therefore important to develop a suitable theoretical framework for understanding the non-linear kinetic properties of phages as “self-replicating pharmaceuticals” [9]. To this end, we test a PK/PD model of phage–bacteria interactions against *in vitro* experimental data for a low passage strain of *Campylobacter jejuni* together with a virulent phage active against this strain.

There are several reasons why a *C. jejuni*-phage system is well-suited for testing how PK/PD models fare against *in vitro* data. First, as a human pathogen *C. jejuni* ranks amongst the major causes of infective gastroenteritis (campylobacteriosis) in the UK, USA and many other countries, and thus is of considerable public health interest [12]–[14]. Second, campylobacteriosis is a zoonotic disease, most commonly contracted following the consumption of contaminated foodstuffs; phage therapy of poultry prior to slaughter, or of meat prior to packaging, could potentially prevent campylobacters from entering the food chain [15]–[17]. Phage therapy against *C. jejuni* is therefore not only important in human medicine but is also relevant to agricultural and veterinary applications. Third, population dynamics of *C. jejuni* cultures are similar to those of many other pathogenic bacteria, including the rise of resistant bacteria following inoculation of susceptible populations with phages. Resistance to phages readily arises in susceptible *in vitro* cultures for most (if not all) kinds of bacteria, and for *C. jejuni* has also been observed to arise *in vivo* in poultry [18]. The *C. jejuni*-phage system is therefore of broad relevance to the nascent field of phage therapeutics.

In this paper we demonstrate the existence of threshold phenomena in experimental data from an *in vitro C. jejuni*-phage system, and illustrate how an understanding of these thresholds will be important in developing practical *in vivo* phage therapies. We focus on estimating the key biological parameters which control the threshold levels, and ask whether these parameters might vary between cultures according to dosage of phage treatments or otherwise between different experimental contexts. We also focus on the role of resistant bacteria. Although resistance to phages has long been an area of interest in basic phage biology and ecology, it is rarely addressed in detail in phage therapy studies despite being a major issue for practical phage therapeutics [19]. Although the characteristics of *in vitro* phage–bacteria interactions are likely to differ from those observed *in vivo*, we view the testing of models of *in vitro* phage therapy to be a necessary first step in understanding how PK/PD theory of phage therapy can be applied in clinical and other *in vivo* settings.

## Materials and Methods

In this section we first discuss the theory behind our model, then the experimental materials and methods and finally the statistical methodology by which we fit the model to the data.

### Theory of phage–bacteria interactions

In phage therapy, the pharmacokinetics and pharmacodynamics (respectively the effect of dosage on phage concentration and the therapeutic effect of the phage) cannot be fully separated. The pharmacokinetics and pharmacodynamics are fundamentally interrelated because phages spread throughout bacterial populations much like epidemics spreading through macro-biological populations: infecting susceptible bacterial cells, reproducing and subsequently infecting other susceptible cells. As with epidemics, the rate of phage growth is dependent on the host population, in that the phage population can only increase when the bacterial concentration is sufficiently high. Wiggins and Alexander [20] investigated the existence of such a threshold bacterial concentration using experimental methods, finding that concentrations of around 10^4^ colony-forming units (CFU) mL^−1^ were required for phage growth on a range of bacterial hosts. Payne and Jansen [9] later derived a formula for this threshold using a mathematical model of phage–bacteria interactions and termed it the *proliferation threshold*: the concentration that the bacterial population must exceed in order for the total phage numbers to increase. Likewise, there is a critical threshold in the phage concentration, the *inundation threshold*, which is the minimum phage concentration above which the bacterial population declines. The inundation threshold has parallels in antibiotic therapy, but the proliferation threshold is unique to self-replicating antimicrobial agents. Unlike previous authors who have compared or fitted models to data for phage–bacteria interactions [11], [21]–[26], we focus on fitting the two thresholds identified above and investigate whether single values for these thresholds adequately explain the dynamics of the phage and bacterial populations over a range of starting conditions.

It is also useful to introduce the concepts of active and passive therapy. *Active* therapy requires ongoing replication of phage in order that the phage concentration reaches or is maintained at levels sufficient to control the bacteria; *passive* therapy is when the initial dose and primary infection is by itself sufficient to reduce bacterial numbers. The two modes are not mutually exclusive and both can occur in the same treatment, for example where the initial phage dose is large enough to suppress the bacterial population and is maintained at that level by phage replication, but it is useful to separate them conceptually. To understand the basic kinetic properties of phage therapy one must appreciate that active therapy can occur only when the concentration of bacteria exceeds the proliferation threshold, and passive therapy can occur only when the initial concentration of phage exceeds the inundation threshold [27].

#### A kinetic model

It is reasonable to assume that in well-mixed experimental systems, interactions between phage particles and bacterial cells occur such that the rate of reaction between any two species is proportional to the product of their concentrations (i.e. mass-action kinetics). These assumptions lead to a model similar to that of Payne and Jansen [9]. That model, however, only focused on the dynamics of phage infection during the initial period of exponential growth of bacteria, and therefore did not include bacterial resistance to phages. In order to extend the analysis of phage–bacteria interactions beyond this short time-scale, we allow that some bacteria may become insensitive to infection, for example by acquiring resistance to the phage. Dealing with resistance is a problem that will be central to the design of phage therapies, because resistant mutants are highly likely to be present in bacterial populations of any size much greater than the inverse of the mutation rate. Our approach yields a system of delay differential equations, where the concentrations of susceptible and resistant bacteria, infected cells and free phage particles at time *t* are *S*, *R*, *I* and *V*, respectively:
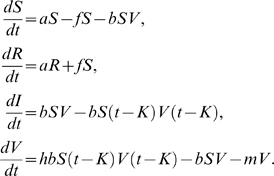
(1)


Here, both susceptible and resistant bacteria grow asynchronously at a constant rate *a* per cell, which takes into account both replication and phage-independent death. Susceptible bacteria may give rise to resistant mutants with probability *f* /*a* per susceptible cell per generation, and thus *f* is the rate of mutation. Infection of susceptible bacteria as a result of the binding of free phage particles to bacterial cells and successful infection is treated as if it occurs according to the principle of mass action, with rate parameter *b*. After a fixed latent period *K*, infected cells are lysed, at which time an average of *h* new phage particles are released into the environment per infected cell. Thus, the dynamics at time *t* depend not only on the concentrations of phages and bacteria at time *t*, but also on the concentrations of free phage particles and susceptible bacteria *K* units of time in the past, as represented by the terms *S*(*t*–*K*) and *V*(*t*–*K*). These new phage particles repeat the same life-cycle or are lost to the system by a process of random degradation, occurring at rate *m*. The parameters are summarised in Table 1.

**Table 1:** Definitions of model parameters for Equation 1.

Parameter	Symbol	Units
growth rate of bacteria	*a*	h^−1^
binding rate of phages to susceptible bacteria	*b*	mL CFU^−1^ h^−1^
mutation rate of bacteria	*f*	h^−1^
latent period (time between infection and lysis)	*K*	h
burst size at lysis	*h*	PFU
phage decay rate due to thermodynamic and other effects	*m*	h^−1^
phage concentration above which susceptible bacteria decline	*V_I_*	PFU mL^−1^
bacterial conc. above which phage replication exceeds phage loss	*S_P_*	CFU mL^−1^

Fitted values for these parameters are given in Table 2 and Table 3.

In this formulation, resistant bacteria are assumed to be totally resistant (and so there is no term for interaction with phages) and resistance cannot be lost once acquired. We also assume that resources are sufficiently plentiful for there to be no competition between susceptible and resistant bacteria. Hence, the concentration of resistant bacteria is driven by, but does not affect the concentration of, susceptible bacteria; consequently the presence of resistant bacteria has no effect on the therapeutic action of phages on susceptible cells (although it may affect the overall therapeutic outcome).

#### Phage therapy

From the equation for *dS/dt*, the concentration of susceptible bacteria can only decline if the concentration of free phages exceeds the inundation threshold *V_I_*, where
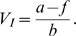
(2)


Thus if the initial concentration is above this inundation threshold then active replication of phages is not essential to therapy, and therapy can then be considered ‘passive’ in this sense. In any case, it is a precondition of therapeutic effect that the phage concentration eventually exceeds the inundation threshold *V_I_*. Active therapy can then proceed only if the total number of phages (free virions plus the phages that are latent in infected cells) is increasing. This occurs only when the phages gained through replication can replace those lost to degradation, or equivalently, whenever the expected number of progeny produced by each phage is greater than 1. The expected number of progeny per virion (also known as the basic reproductive number) can be calculated from Equation 1, resulting in the condition that the total number of phages increases if *hbS/*(*m*+*bS*)>1. Solving this for the concentration of susceptible bacteria *S*, we find that the total phage concentration increases when *S* exceeds the proliferation threshold,
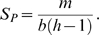
(3)


Except for the mutation rate *f*, these thresholds are the same as those from Payne and Jansen [10], because they relate only to the concentration of susceptible bacteria. While therapeutic action is predicated on the phage concentration eventually exceeding the inundation threshold *V_I_*, the initial dosage is not necessarily important as long as the bacterial concentration grows above the proliferation threshold *S_P_* before too many phage particles degrade. Thereafter, active replication of phages will bring the phage concentration up above *V_I_* and the phage will have a therapeutic effect.

The inundation and proliferation thresholds are of considerable practical importance to phage therapy. Not only do they delimit active and passive phage therapies and characterise the peculiar properties of phages as antimicrobial agents, but they can also be used to prescribe dosage and timing of a phage treatment so as to achieve the greatest therapeutic effect [10],[27]. One could infer the physical parameters of phage–bacteria interaction (binding rate; bacterial growth rate; latent period prior to lysis; burst size; phage degradation rate) from data and use the model Equation 1 to predict the outcomes of the joint population dynamics *in vitro*. However, the inundation threshold *V_I_* and the proliferation threshold *S_P_* encapsulate the primary information about the therapeutic effect of a phage treatment. To help gain insight into these thresholds we re-parameterise the model Equation 1 in terms of *V_I_* and *S_P_*. Together, these parameters can replace the binding rate *b* and the burst size *h*, which are difficult to estimate directly and are also likely to differ *in vivo*, when phage treatments are applied outside the laboratory, from those that are found *in vitro*. (The parameters *b* and *h* can of course be found from *V_I_*, *S_P_*, *a*, *f* and *m* by rearranging Equation 2 and Equation 3.) These two threshold parameters are also summarised in Table 1.

### Experimental methods

#### Propagation and enumeration of campylobacters and bacteriophage

*Campylobacter jejuni* strains were routinely propagated on blood agar base no. 2 (Oxoid Ltd., Basingstoke, United Kingdom) supplemented with 5% defibrinated horse blood (Oxoid) for 24 h under microaerobic conditions (5% O_2_, 10% CO_2_, 85% N_2_) at 42°C. *C. jejuni* were enumerated using *Campylobacter* blood-free selective agar base (Oxoid). *Campylobacter* bacteriophage CP8 [16] was routinely propagated on strain NCTC 12662 (phage type 14; PT 14), however, bacteriophages from interaction experiments and bacteriophage decay experiments were enumerated on host lawns of *C. jejuni* strain HPC5 [16].

#### Bacteriophage decay experiment

To determine the rate of decay of free phage in the absence of a host, bacteriophage CP8 was added to 50 mL of nutrient broth no. 2 (Oxoid) in 100 mL conical flasks and incubated at 42°C under microaerobic conditions with shaking at 100 rpm. Samples were taken for up to 168 h and bacteriophages enumerated as above.

#### *Campylobacter* and bacteriophage binding experiments

To assess the latent period and burst size of a single round of phage replication, *Campylobacter jejuni* strain GIIC8 was grown and inoculated into 50 mL of nutrient broth no. 2 (Oxoid) in 100 mL conical flasks as previously described. The triplicate cultures were grown to late log phase, approximately 8.3 log_10_ CFU mL^−1^, at which point CP8 was added to an approximate MOI of 0.01. Incubation with shaking was continued and samples of the culture removed at various time points, up to 2 h post phage addition. Samples were filtered to remove *Campylobacter* cells and bound phages. Unbound ‘free’ phages were titrated as described previously.

#### *Campylobacter* and bacteriophage interaction experiments

*Campylobacter jejuni* strain GIIC8 [16] was grown on blood agar plates for 24 h as described above. Cells were collected into phosphate buffered saline and OD_600_ readings taken. GIIC8 was added to 50 mL of nutrient broth no. 2 (Oxoid) in 100 mL conical flasks to a final concentration of 5 log_10_ colony-forming units (CFU) mL^−1^ and incubated at 42°C under microaerobic conditions with shaking at 100 rpm. At 2 h post inoculation, bacteriophage CP8 was added to a final concentration of 5 log_10_ plaque-forming units (PFU) mL^−1^, 6 log_10_ PFU mL^−1^ or 7 log_10_ PFU mL^−1^ to give approximate multiplicities of infection (MOI) of 1, 10 or 100. Samples were taken every 2 h for 24 h. Each aliquot was serially diluted and campylobacters enumerated as described above. Bacteriophages were enumerated as above following passage of the cell suspension through a 0.2 *µ*m Minisart filter (Sartorius AG, Göttingen, Germany) to remove the *Campylobacter* cells.

### Statistical methods

Counts of bacterial colonies or phage plaques were assumed to be Poisson distributed with a mean count given by the true concentrations of bacteria or phages divided by a known dilution factor. Model parameters were optimised for the phage decay data (phage decay rate *m*) in a generalised linear model (GLM) fitted by iteratively re-weighted least squares [28], with initial parameter estimates chosen automatically by the glm algorithm in the R statistical computing environment.

For the binding assay (all parameters except the mutation rate *f*) and interaction data (all parameters), the non-linear model given by Equation1 was fitted using a generalised non-linear model (GNLM). Starting values for initial phage and bacterial concentrations were estimated for each culture as the mean concentrations at time 2 h. Initial parameter values were selected by hand and then estimated parameters were found using a variant of simulated annealing [29] followed by the ‘L-BFGS-B’ quasi-Newton algorithm [30]. Two optimization algorithms were used to attempt to ensure that the fitted parameters did not get stuck at a spurious local optimum. J.K. Lindsey's gnlm package for R was modified to use the function optim, which implements above-mentioned optimisation routines, and was used to perform the fitting procedure [31].

In the case of the interaction data, the model was re-parameterised in order to focus on the aspects of most relevance to phage therapy. The proliferation threshold *S_P_* and the inundation threshold *V_I_* together replaced the binding rate *b* and the burst size *h* according to Equation 2 and Equation 3. Approximate starting parameter values were chosen as follows. Rough starting values for the threshold parameters and for the bacterial growth rate *a* were obtained by inspection of the plotted data. The starting values of the phage decay rate *m* were taken from the analysis of the phage decay data, and the starting values for the latent period *K* and burst size *h* were obtained from the analysis of the binding assay data. A mutation rate *f* of 10^−6^ h^−1^ was selected as a starting value by inspecting the data and extrapolating back to obtain the approximate ratio of resistant to susceptible cells in the exponential phase; this rate is faster than would be typical for point mutations. Observations at time 0 h were omitted because they suggested an initial lag phase prior to exponential growth of bacteria that is beyond the scope of the model Equation 1. Data for the two cultures in which phages were not present were additionally truncated at 12 h to confine observations to the exponential period of growth; in these cultures the bacterial populations appeared to enter stationary phase shortly after 12 h.

Although in principle it is possible to examine the fit of any model by an approximate test of the significance of the model derived from analysis of deviance tables, and to compare models by use of likelihood ratio tests, in a statistical context models like Equation 1 may violate the assumptions of likelihood ratio methods and admit such a wide range of population dynamics that the overall model is unlikely to be rejected [22]. Instead, we compare model variants by use of the Akaike Information Criterion (AIC) [32]–[33], a quantity derived from information theory that may be used to compare models with different numbers of parameters. (The AIC is defined as the negative of twice the log-likelihood of the fitted model, plus a penalty defined as twice the number of free parameters in the model. Values of the AIC for competing models can be compared to judge which provides a better fit: lower values of the AIC indicate a closer fit to the data.)

## Results

### Bacteriophage decay experiment

**Figure 1:**
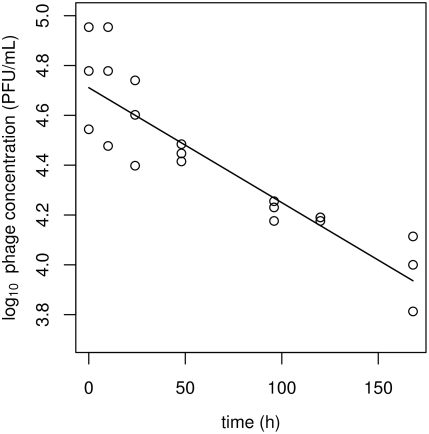
Phage decay in the culture medium in the absence of hosts. The plot shows data from one of the three cultures in the phage decay experiment, with observed concentrations in log_10_ PFU mL^−1^ (points) and the fitted curve log_10_ *V*(*t*) = log_10_ *V*_0_−*mt* (line). The phage concentration declines linearly on log-linear axes, indicating approximately exponential decay with estimated rate 1.062×10^−2^ h^−1^.

The phage decay experiments suggest that in the absence of bacteria, phage virions degrade at a roughly constant rate, such that the concentration of phages declines linearly on a log-linear plot (Figure 1). The rate of phage degradation fitted by GLM is *m* = 1.062×10^−2^ h^−1^ (95% CI 0.961×10^−2^–1.165×10^−2^), with fitted initial phage concentrations of between 5.01×10^5^ and 6.32×10^5^ PFU mL^−1^. The null deviance (including distinct intercept terms for each culture but no decay term) is 514.79 on 60 degrees of freedom, and the residual deviance for the phage decay GLM is 80.01 on 59 degrees of freedom.

### *Campylobacter* and bacteriophage binding experiments

**Figure 2:**
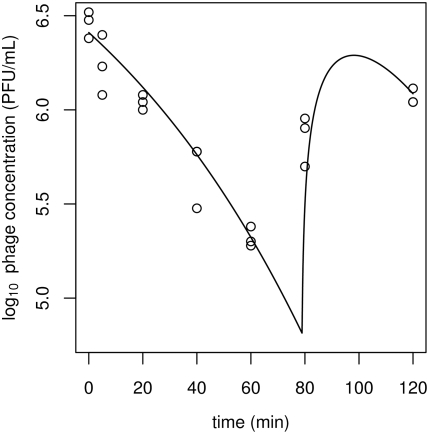
Binding of phages to bacterial hosts and the subsequent release of phage at lysis of infected cells. The plot shows data from one of the three cultures in the binding assay, with observed concentrations of free phages in log_10_ PFU mL^−1^ (points) and the fitted curve *V*(*t*) (line), derived from Equation 1. Lysis of infected hosts is well synchronised at approximately 1.3 hours, although this is exaggerated by the fitted curve. The phage concentration does not appear to increase, which is consistent with the low estimate for the burst size (approximately equal to 2 virions).

Binding assays show a steep decline in the concentration of free phages due largely to binding of free phages to susceptible bacteria, followed by a rapid, somewhat synchronised increase in this concentration as a second generation of phages is released by lysis of infected cells (Figure 2). This series of observations of phage concentrations comprises a single round of phage replication. The fitted parameters of interest are the latent period *K* = 1.312 h and the burst size *h* = 1.957 virions per cell. Other fitted parameters are ‘nuisance’ parameters in the analysis of this data, because they are closely correlated and hence are liable to be inaccurate. Their values are the bacterial growth rate *a* = 0.272 h^−1^, the binding rate *b* = 8.028×10^−8^ h^−1^ and the phage decay rate *m* = 1.032×10^−2^ h^−1^. Fitted initial phage concentrations varied between 2.76×10^6^ and 2.84×10^6^ PFU mL^−1^, while fitted initial bacterial concentrations varied between 2.68×10^7^ and 3.03×10^7^ CFU mL^−1^.

### *Campylobacter* and bacteriophage interaction experiments

**Figure 3:**
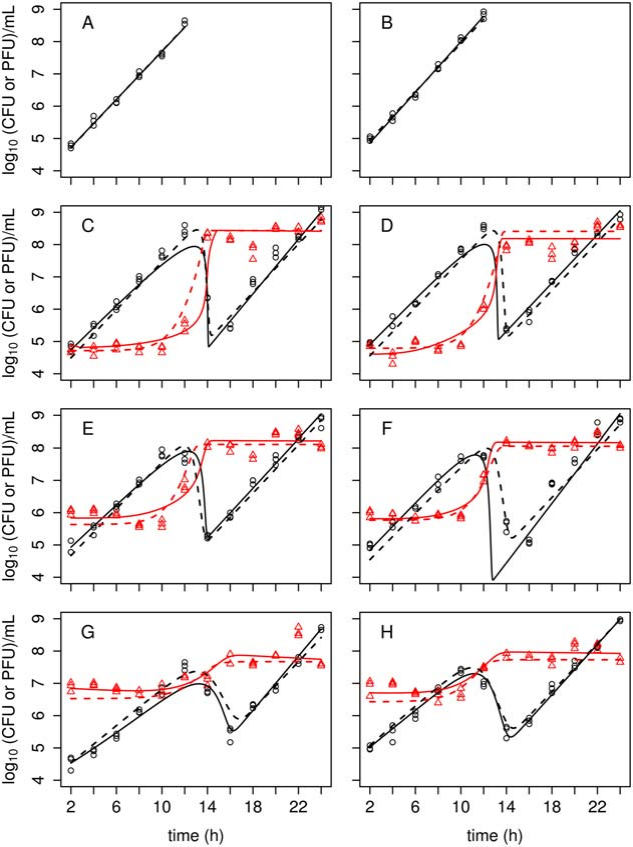
Data from interaction experiments together with fitted curves obtained by maximum likelihood estimation. Concentrations of *C. jejuni* (circles; black) and phage (triangles; red) vary together in a way consistent with the threshold theory of phage-bacterial interactions. Fitted models in which parameters are either common to all (dashed) or allowed to vary between cultures (solid). Panels (A–H) correspond to cultures 1–8, respectively, with fitted parameters as given in Table 2 and Table 3.

Data from phage–bacteria interaction experiments show a regular pattern: exponential growth of bacteria followed after some time by rapid phage proliferation; a sudden crash in the bacterial population and the slowing of phage growth; finally, the resurgence of exponentially-growing bacteria that do not appear to be susceptible to the phage (Figure 3). Overall, the model Equation 1 provides a good fit to these data, both where model parameters are treated as common to all cultures (the ‘common-parameter’ model; Table 2) and where they are allowed to vary between cultures (the ‘varying-parameter’ model; Table 3). These fitted models match very well to the observed phage and bacterial concentrations up to the end of the crash in the concentration of susceptible bacteria and the beginning of the growth of (apparently) resistant bacteria around 14–16 h (Figure 3). Beyond that time there is a pattern of exponentially-growing bacteria despite a high phage concentration. This is reflected in the model, but there is a greater discrepancy between the fitted and observed values. The difference between the AIC values for the ‘varying-parameter’ model (AIC≈4948.1) and the ‘common-parameter’ model (AIC≈5765.5) is very large (≈817.4), indicating that the former provides a much better fit to the data.

**Table 2:** Model parameters from Equation 1 where parameters are common to all cultures, fitted to data from the interaction experiments.

Fitted parameters (common-parameter model)								
	*a*	log_10_ *f*	log_10_ *b*	*h*	*K*	*m*	log_10_ *V_I_*	log_10_ *S_P_*
	0.8604	−4.9875	−7.2310	1.5501	0.0944	0.0003	7.1657	4.0309
**Initial conditions**								
**MOI**	0		1		10		100	
**Culture**	*1*	*2*	*3*	*4*	*5*	*6*	*7*	*8*
log_10_ *S*_0_	4.7142	4.9509	4.4892	4.5580	4.6689	4.5432	4.5428	5.0803
log_10_ *V*_0_	—	—	4.7154	4.7903	5.6328	5.7673	6.5282	6.4301

Estimates were obtained by maximum likelihood as described in the text; parameter notation is as in Table 1. The total AIC for this model is 5765.540.

**Table 3:** Model parameters from Equation 1 where parameters are allowed to vary between cultures, fitted to data from the interaction experiments.

Model parameters (varying-parameter model)											
MOI	Culture	Initial conditions		Parameters							
		log_10_ *S*_0_	log_10_ *V*_0_	*a*	log_10_ *f*	log_10_ *b*	*h*	*K*	*m*	log_10_ *V_I_*	log_10_ *S_P_*
0	*1*	4.726	—	0.8545	—	—	—	—	—	—	—
	*2*	4.862	—	0.9028	—	—	—	—	—	—	—
1	*3*	4.742	4.888	0.9738	−5.675	−5.561	2.080	0.8563	0.0067	5.550	3.353
	*4*	4.906	4.627	0.8608	−4.940	−5.931	1.624	0.4314	0.0009	5.866	3.096
10	*5*	4.929	5.837	0.8781	−5.017	−6.738	1.795	0.5563	0.0022	6.682	4.190
	*6*	4.844	5.821	1.0614	−6.508	−6.468	1.840	0.6005	0.0033	6.494	4.063
100	*7*	4.531	6.844	0.9679	−5.459	−7.180	3.023	0.9168	0.0416	7.166	5.493
	*8*	5.024	6.707	0.8975	−5.172	−7.322	2.366	0.5004	0.0107	7.275	5.218

Estimates were obtained by maximum likelihood as described in the text; parameter notation is as in Table 1. The total AIC for this model is 4948.075. There is a strong positive relationship between the logarithm of the initial phage concentration and the logarithms of the inundation threshold *V_I_* and proliferation threshold *S_P_*, which together indicate dose-dependence of the binding rate *b*.

The roles of some parameters in the model are of particular interest, and were examined by re-fitting the common-parameter model with these parameters set to 0. Some form of resistance to phages is an essential component of any model that can fit the pattern of bacterial and phage growth followed by the crash and resurgence of the bacterial population observed for this system. A variant of the common-parameter model without resistance (i.e. with mutation rate *f* = 0) is unable to fit the data after the steep decline in the bacterial concentration and provides a very poor fit over the entire time series (AIC≈27571). Conversely, the common-parameter model has an unrealistically small fitted value of the latent period *K* between infection and lysis. Nonetheless, the non-zero latent period of the common-parameter model gives a substantially better fit, compared to the model with *K* = 0 (AIC≈6139).

## Discussion

### Thresholds

The inundation and proliferation thresholds help define two modes of phage therapy (passive and active) as well as the success or failure thereof, and can be used to gain insight into the kind of information required to develop practical treatment protocols. Our time series clearly show an inundation threshold; the bacteria grow readily in the presence of a virulent phage until the phage reaches a critical concentration beyond which susceptible bacteria rapidly become infected. The effect of crossing a proliferation threshold is more difficult to observe directly, in part due to the fact that it is a threshold for the increase of total phage but not necessarily for free phage. For example, in the binding assays (Figure 2), although the bacterial concentration is very high the concentration of free phage does not appear to increase relative to the initial concentration at the first round of replication. The interaction experiments (Figure 3) do, however, show a clear change from a slow decline to a rapid increase in the phage concentrations after a period that appears to be independent of the initial phage concentration and is much longer than the delay between infection and lysis of individual cells. One could attempt to estimate the proliferation threshold from time series data ‘by eye’, without recourse to complicated statistical methods. Such estimates would be very imprecise, partly because they depend on the delay in the response of the phage population to a change in the bacterial population. We therefore used a formal statistical procedure to estimate the values of both the inundation and proliferation thresholds, using visual estimates as starting values only. We have shown that our model provides an excellent fit to the data, and this is consistent with the threshold-based view of phage–bacteria interactions.

The values of these thresholds and other model parameters will differ *in vivo*, and indeed in other *in vitro* contexts. Nonetheless, our results also have implications for future experimental and clinical protocols for phage therapy. Two predictions regarding the dose of a phage inoculum can be derived from the model that are also reflected in the interaction data presented above. First, at lower doses the response of a bacterial population of a given size to a phage will vary little with the dosage of a phage inoculum. The phage concentration will not exhibit net growth unless the host concentration is above the appropriate proliferation threshold, and when this occurs phage proliferation becomes relatively rapid until the phage concentration reaches the inundation threshold and begins to suppress the host population (phage therapy in an active mode). Thus a wide range of initial phage doses should suppress the bacterial population at roughly the same time; this might be particularly important where the timing of phage therapy is relevant, such as in agricultural or food safety contexts. Second, at higher doses—those close to or greater than the inundation threshold—the effect of a phage on the bacterial population will change rapidly as the dosage increases. As the initial phage concentration is increased beyond the inundation threshold, the growth rate of the bacterial population will quickly shift to an exponential decline. In cases where the phage concentration starts substantially above the inundation threshold, phages can be expected to infect all susceptible host cells in a relatively short period of time (phage therapy in a passive mode).

### Resistant bacteria

Although molecular and evolutionary questions relating to phage resistance have been addressed in detail in the past (reviewed in [19]), as far as we are aware this is the first phage therapy study in which models that incorporate acquisition of resistance have been fitted directly to *in vitro* time series data. The advantage of this approach to considering the rise of resistant cells in a phage therapy context is that it provides a more practical assessment of the role that resistant cells might play. The threshold theory of phage therapy implies that the growth of resistant bacteria and the physical properties required for apparent resistance are very sensitive to environmental conditions and phage concentrations; in some cases, cells that are only slightly less susceptible than the wild-type can grow when the wild-type cannot [19]. Thus, it is nearly impossible to directly compare the outcomes of batch cultures (let alone *in vivo* or other practical trials of phage therapies) and other tests where the purpose of the comparison is to understand the role of resistance in phage therapies. Although we fit the rate at which resistant cells arise by mutation from a susceptible population, the definition of ‘resistance’ is specific to the conditions that persist in these cultures. Instead, the focus here is on understanding the qualitative role that resistant bacteria may play in phage therapy.

A particular feature of the interaction data is the resurgence of bacteria after the phage-induced crash of the susceptible population. Some form of resistance—whether due to point mutation or other processes—seems necessary to explain this pattern, and it appears to be heritable or transmissible because the concentration of apparently-resistant bacteria continues to grow in the presence of high concentrations of phages. These resistant bacteria exhibit several other interesting features. First and, from a therapeutic perspective, most importantly, the rate of growth of the resistant bacteria is approximately the same as that of the original, susceptible population, indicating that there is little or no fitness disadvantage to the apparently-resistant cells under the conditions of these *in vitro* experiments. Previous studies have found a reduction in fitness of phage-insensitive campylobacters in the gut of broiler chickens when phages are absent, evidenced by a decrease in colonization efficiency [18]. Although our result might be specific to the *in vitro* context of our experiments, it is unreasonable to assume that significant fitness disadvantages will be incurred under all environmental conditions. Whether resistant cells have reduced pathogenicity is an important question for future study. Second, in each culture from the interaction experiments that includes the phage, the resistant bacteria appear at roughly the same time, although there are too few cultures to expect much variation in this quantity and in any case all cultures were inoculated with samples from the same source population. The fitted rate of mutation to resistance, *f*, is of the order of 10^−5^ h^−1^, which is relatively fast, but might be consistent with hyper-mutability of some individuals, with more complex genetic (or epigenetic) phenomena such as recombination (known to be a mechanism by which resistance is acquired in other strains of *C. jejuni*; [18]) or phase variation [34]–[36], or with a combination of different types of resistance. Tracing the resistant population back to the beginning of the experiment, it is apparent that an alternative explanation is that a very small number of resistant cells were present in each culture at the start of the experiment, although this also suggests a high rate of mutation to resistance that might be explained as above.

The assumption that bacteria are either susceptible or totally resistant is adequate to explain most of the variation in the data, but there are some discrepancies. One of the chief discrepancies is that the phage concentration does not simply reach a peak then slowly decline, as suggested by the fitted curve. Instead, there appears to be a second peak, appearing some hours after the first (Figure 3, panels C–H, at around 20 hours). It is possible that this phenomenon is artefactual, but it is beyond the scope of the simple model to predict such dynamics. Possible interpretations of this phenomenon include more than one type of resistant sub-population, the mutation of phages to infect resistant bacteria [19],[37], or possible coexistence dynamics of phages and susceptible bacteria [38]–[40]. Critically, the threshold-based model implies that even a partially-resistant sub-population of bacteria could be responsible for the recovery of the bacterial concentration, provided only that its inundation threshold is sufficiently larger than that of the original susceptible population and than the phage concentration following the crash. The possibility that such partially-resistant cells might be responsible for resurgence of bacterial populations highlights the need for a threshold-based understanding of phage–bacteria interactions for phage therapy.

Cells that are resistant to a phage should be expected to be found with a high probability in bacterial populations above a certain size, and phage therapy is by its nature a strongly selective treatment. Therefore, resistance must be taken into account in both theoretical and empirical studies of phage therapies [19],[37]. This is not to say that the likelihood of resistance is an insurmountable problem for phage therapy, even if in some cases it might not be possible to avoid it. In the context of *in vivo* control of campylobacters in poultry, achieving a reduction in *C. jejuni* load at the time of slaughter could reduce campylobacter numbers on retail poultry products [16] and hence the incidence of human campylobacteriosis [41]. Optimising the dosage and timing of the phage treatment would then be sufficient to avoid problems with resistant bacteria. In other contexts, it may be necessary to take measures to suppress resistant bacteria, for example by use of a cocktail of phages that bind to different receptors.

### Differences between cultures

The common-parameter model (Table 2), in which the model parameters are assumed to be the same for all cultures, appears to provide a good fit to the data (Figure 3). But by allowing model parameters to vary between cultures the fit is substantially improved. The purpose of allowing these parameters to differ is to investigate how the model of Equation 1 might deviate from the true dynamics of the *C. jejuni*-phage system. The varying-parameter model shows a specific trend in the fitted values of the inundation threshold *V_I_* and the proliferation threshold *S_P_* (Table 3). As the initial phage concentration increases, fitted values of both thresholds also increase; the relationships are roughly linear in the logarithms of each of these parameters. Under the assumptions of the model, this implies that the binding rate *b* declines with increasing initial concentration of phages; there are no other strong relationships between fitted parameters (Table 3), although the burst size *h* and the phage decay rate *m* are slightly larger in cultures 7 and 8, which have the highest initial phage concentrations. The fitted phage decay rates are otherwise similar to those found in the decay experiment and binding assay. A similar trend in the binding rate with respect to initial phage concentration was observed by Mudgal *et al*. [24] in interactions between phage and *Leuconostoc* species, which are used in the fermentation of sauerkraut (and for which phages are a pest, rather than a therapeutic agent). It is important to note that this trend in the fitted values of *b* may either represent true differences between cultures in the effective binding rates, or mask other differences between cultures that are not included in the model but which are best approximated by the fitting algorithm with a change in *b*.

A wide range of phenomena might plausibly explain the decline in the fitted binding rate *b* as the initial concentration of the phage increases. Such explanations include restriction-modification systems [42] or other responses by the bacterial population to the presence of phages, the contribution to the loss of free phages due to unproductive binding of phage particles to resistant bacteria or debris from lysis [38] or the superinfection of already-infected cells and possible subsequent lysis inhibition [43]. These phenomena could play a substantial role in the success or otherwise of phage therapies but are difficult to assess from time series data such as ours. Although their microbiological and ecological implications are well-studied, more work is needed to assess their practical importance for phage therapies.

Another possibility is phenotypic diversity in the phage or host populations, due to genotypic or physiological differences between individuals in the initial populations and subsequent selective effects. For instance, it is possible that phages from the original stock are less effective at binding to the GIIC8 strain of *C. jejuni*. Alternatively, the phage preparation may contain defective interfering particles, which are unable to replicate by themselves and can interfere with the replication of functional viruses when they co-infect [44], or other non-infective virus particles that could competitively exclude functional phage particles from their binding sites on susceptible cells. In these cases, the larger the size of the phage inoculum the greater the proportion of the original stock that makes up the phage concentration at any given time and the lower the effective binding rate.

The inundation threshold may vary if there is heritable variation in the binding rate or in the susceptibility to successful infection after binding. In this case, a large initial phage concentration could selectively enrich the more-resistant bacteria. Consequently, the average susceptibility of the bacterial population at its peak concentration will be lower than would otherwise be the case, and so the apparent inundation threshold will be higher. It is difficult, however, to assess the magnitudes of such effects in the absence of detailed information about the genetic diversity of the phage populations.

Whatever the underlying biological mechanisms, it is plausible that trends in the inundation and proliferation thresholds or the binding rate could also play a role *in vivo*. If trends similar to those we have found here were found *in vivo*, it might imply that there is a non-trivial optimal dose, yielding the fastest and most effective suppression of the bacterial population, when a phage therapy is administered in an active mode. Further, the dose required to inundate bacteria with phages in a passive therapy might be inflated, relative to the concentration that suppresses bacteria following active proliferation, due to increased inundation thresholds. Overall, aspiring phage therapists should be aware that such trends are consistent with *in vitro* data, and that dose-dependent changes in threshold phenomena might be observed in therapeutic contexts.

### The latent period

A comparison may be made between the interaction experiments (which are designed to show the overall dynamics of phage–bacteria interactions) and the phage decay experiment and the binding assay (which focus on particular parameters). The latent period *K* is of particular interest here. The value of *K* fitted to the data from the binding assay is approximately 1.3 h (corresponding to the minimum concentration of the fitted curve; Figure 2), while the values of *K* fitted to the data from the interaction experiment are between about 0.5 h and 0.9 h where the parameters may vary between cultures, and less than 0.1 h for the common-parameter fitted model. There is no apparent relationship between the values of *K* in the varying-parameter model and the initial concentration *V*_0_ of phages, but there does appear to be a relationship with the initial concentration *S*_0_ of bacteria. But, since the fitted values differ substantially from the quantity which is more directly observed in the binding assay, it seems unlikely that there is a biological reason for such a close relationship between the fitted values of the latent period and the initial bacterial concentration. In any case, all of these fitted values of the latent period *K* are much shorter than the period before rapid proliferation of phage observed in the interaction data (Figure 3, panels C–H), supporting the interpretation of the delay before rapid phage growth as evidence of a proliferation threshold.

Although the latent period plays an important role in the life cycle of individual phage particles, the model Equation1 suggests that the latent period may not always be as important as other parameters in determining the overall dynamics of phage–bacteria interactions. The phage life-cycle may be divided into the time spent between hosts, ending when the free phage binds to a susceptible cell, and the latent period between infection and lysis. The time spent between hosts has mean duration 1/*bS*, for binding rate *b* and concentration *S* of susceptible bacteria, while the latent period between infection and lysis is given by the parameter *K*. Because *b* is very small, only when the population of susceptible bacteria is large will the latent period be an appreciable fraction of the total life-cycle duration (1/*bS*+*K*). For values of *b* around 10^−6^ ml CFU^−1^ h^−1^ and *K* equal to about 1 h, the concentration *S* of susceptible bacteria would have to be close to 10^6^ CFU mL^−1^ in order for the latent period to contribute a large portion of the total life-cycle time. But the observed concentration of susceptible bacteria is only in this range for a fraction of the duration of the interaction experiment before the bacterial population crashes (Figure 3), and thus this experiment may contain relatively little information about the latent period. Over most of the time series of interaction data, the phage dynamics are primarily determined by the binding rate *b*, the burst size *h* and the decay rate *m*, but not the latent period *K*. Moreover, because the phage growth rate is also determined by the burst size *h*, the fitting procedure can trade *K* off against *h* or other parameters to obtain a good fit even if *K* is inaccurate. (This is a statistical trade-off; there may also be a fitness trade-off for phage between latent period and burst size [45] but we do not address such evolutionary issues here.) We tested the capability of the model to fit the data when the latent period *K* was set to 0. Although a non-zero latent period provides a substantially better fit according to the AICs, there is less of a difference between the main common-parameter model and its *K* = 0 variant than there is between the common-parameter and varying-parameter models. This further indicates that, at least in some cases, the dynamics of phages and bacteria are not strongly sensitive to the latent period.

### Conclusions

Despite their long history, phage therapies are still far behind chemical antibiotic therapies in both theory and practice. If phage preparations are to fulfill their promise as self-replicating antimicrobials, an understanding of the kinetics unique to phage–bacteria interactions must be developed and applied in a therapeutic context. Recent years have seen renewed attention given to models of these interactions, and lately to validating predictions from such models with data from a variety of bacterial species and virulent phage strains. It should be anticipated that any simple model for phage–bacteria interactions will not capture all aspects of a particular combination of phage and bacterial strains, but it is important that models used in designing a phage treatment do provide accurate predictions of parameters such as the inundation and proliferation thresholds. To date, much of the study of phage–bacteria interactions has been grounded in pure rather than applied concerns, and recent models have tended to focus more on ecological and evolutionary issues than on the effectiveness of a particular phage treatment in controlling a bacterial population. As we have attempted to do here, these approaches would benefit from being recast in terms relevant to phage therapy in order that they may contribute constructively to advancing therapeutic goals. Models examined in this way and tested against empirical data can then provide considerable insight into therapeutic issues.
